# Freeze–Thaw Damage Evolution of PVA–Fly Ash–Slag Composite Concrete and Low-Dimensional Mapping of Weibull Characteristic Parameters

**DOI:** 10.3390/ma19173767

**Published:** 2026-09-04

**Authors:** Xinyu Liang, Guang Cheng, Jiaqi Zhao, Rui Li

**Affiliations:** School of Electric Power, Civil Engineering and Architecture, Shanxi University, Taiyuan 030031, China; chengguang@sxu.edu.cn (G.C.); zhaojiaqi1@sxu.edu.cn (J.Z.); lirui4617@sxu.edu.cn (R.L.)

**Keywords:** PVA fiber–fly ash–slag powder concrete (PVA-FA-SPC), freeze–thaw damage, Weibull function, low-dimensional empirical model, leave-one-mixture-out cross-validation (LOMO-CV)

## Abstract

To investigate freeze–thaw damage evolution and mix proportion effects in PVA fiber–fly ash–slag powder composite concrete (PVA-FA-SPC), nine composite concrete mixtures were designed using an L_9_(3^3^) orthogonal array at a water to binder ratio of 0.45, with plain concrete serving as the reference, and subjected to 200 rapid freeze–thaw cycles. Freeze–thaw resistance was evaluated using surface deterioration, the mass loss rate, and the relative dynamic elastic modulus. Within the investigated factor levels, PVA fiber volume content exhibited the strongest main effect trend, followed by the total mineral admixture replacement rate and the fly ash to slag powder mass ratio. T20R1:2P0.3, containing 20% total mineral admixture replacement, a fly ash to slag powder mass ratio of 1:2, and 0.3% PVA fiber, showed the best measured performance, retaining a relative dynamic elastic modulus of 79.18% after 200 cycles. A two-parameter Weibull function was used as a phenomenological description of the damage evolution; compared with the classical exponential model, the average RMSE decreased from 0.032 to 0.019 and the average MAPE decreased from 5.07% to 2.98%. Because only nine independent orthogonal mixtures were available for parameter mapping, a common shape parameter of α_0_ = 2.1403 was adopted, and a parsimonious equation for the scale parameter β was selected using the small-sample-corrected Akaike Information Criterion (AICc) together with leave-one-mixture-out cross-validation (LOMO-CV). LOMO-CV yielded R^2^ = 0.886, RMSE = 0.034, MAE = 0.025, and WMAPE = 19.98%; however, T20R1:2P0.3 exhibited a node WMAPE of 72.75%, indicating a local limitation of the reduced order mapping. Three non-orthogonal mixtures within the same material system yielded R^2^ = 0.943, RMSE = 0.026, and WMAPE = 15.36%. The proposed model is therefore intended for local trend analysis and preliminary mix screening within the calibrated material system and parameter range rather than for universal service life prediction.

## 1. Introduction

In cold regions, when concrete is subjected to repeated freeze–thaw cycles, the freezing and expansion of pore water, along with the migration of unfrozen water, generate hydraulic and seepage pressures. These pressures cause microcracks to gradually form, propagate, and connect within the paste, aggregates, and the interface transition zone. Macroscopically, this manifests as surface spalling, mass loss, and a decline in the dynamic elastic modulus [1,2,3,4,5]. Freeze–thaw durability is not only governed by porosity, pore size distribution, the bubble spacing coefficient, and saturation, but is also closely related to the water-to-binder ratio, the composition of the cementitious material, fibers, curing regimens, and freeze–thaw cycles [6,7]. Therefore, mix design effects and their predictive models should be discussed within clearly defined material systems and test conditions.

Fiber reinforcement is a common approach to improving the crack resistance and post-freeze–thaw integrity of cement-based materials. PVA fibers possess high tensile strength, good hydrophilicity, and strong interfacial adhesion to the cement matrix. They can limit the opening and propagation of freeze–thaw-induced microcracks through crack bridging, crack deflection, and stress transfer, thereby delaying stiffness decay and surface spalling [8,9,10,11,12]. Recent experimental results indicate that the improvement in post-freeze–thaw mechanical properties and durability achieved by PVA fibers exhibits significant dosage effects and environmental dependence. Liu et al. [13] observed differential effects of fiber dosage on post-freeze–thaw static and dynamic mechanical properties under extremely low-temperature freeze–thaw conditions; Su et al. [14] and Wan et al. [15] confirmed, in a coupled humid heat–chloride environment and a salt–freeze environment, respectively, that an appropriate amount of PVA fibers can bridge microcracks and improve the pore structure, but an excessive amount of fibers may weaken the benefits due to poor dispersion and additional defects; Wu et al. [16] tested fiber contents ranging from 0% to 0.5% under coupled sulfate erosion and freeze–thaw conditions and found that the 0.3% group exhibited superior overall durability. The optimal fiber content varies across different studies, indicating that the fiber effect is jointly influenced by the matrix composition, fiber geometric characteristics, dispersion state, and environmental conditions; this also serves as the direct basis for setting three volumetric content levels—0.1%, 0.3%, and 0.5%—in this study.

Fly ash and slag powder can modulate the hydration process and pore structure through micro-aggregate filling, pozzolanic reactions, and potential hydraulic reactions. The blended use of these two materials is expected to balance particle gradation, early-stage structure formation, and late-stage densification; however, their freeze–thaw resistance is jointly influenced by the total replacement rate, blending ratio, water to binder ratio, and curing conditions, and does not increase monotonically with the admixture content [17,18,19,20,21,22]. The crack control effect of PVA fibers may synergize with the structural regulation effect of mineral admixtures, or it may be constrained by slurry strength, pore connectivity, and fiber dispersion. For the PVA fiber–fly ash–slag powder ternary system, it is necessary to systematically compare the combined effects of the total replacement rate, ash–slag ratio, and fiber content under the same test conditions.

Freeze–thaw damage typically manifests as a nonlinear process characterized by slow accumulation in the early stages and accelerated development in the middle and late stages. Comparing only the mass loss rate or relative dynamic elastic modulus at the end of a single cycle fails to fully reflect how the damage rate varies across different stages of the cycle. The Weibull function, with its few parameters, clear monotonicity, and adjustable curve shape, has been used to analyze the evolution of freeze–thaw damage in concrete and to determine the critical number of cycles [23,24,25]. In recent years, Cheng et al. [26] established a Weibull freeze–thaw damage model for steel-fiber-reinforced coal gangue concrete using indicators such as the relative dynamic elastic modulus, while Yao et al. [27] constructed a freeze–thaw damage model for wastewater concrete using the relative dynamic elastic modulus as the damage variable. These studies demonstrate that the Weibull function can flexibly characterize the nonlinear degradation processes of various cement-based materials. It should be emphasized that when the damage variable is defined as the average relative dynamic elastic modulus of a finite number of test specimens, the Weibull function is primarily a phenomenological curve expression; its parameters should not be directly interpreted as statistical failure probabilities or engineering lifetimes across different material systems without verification.

Building on single-mixture damage curve fitting, a more demanding task is to relate mix design variables to Weibull characteristic parameters without overfitting a small number of independent mixtures. Previous studies have used multivariate regression or optimization to analyze fiber and mineral admixture effects [28,29,30]; however, the reliability of such empirical models depends strongly on the number of independent mix designs, the candidate-variable structure, and the validation strategy. With a small sample and too many estimated coefficients, an apparently high in-sample fit can coexist with unstable parameters and poor performance for mixtures not used in calibration. The Akaike Information Criterion corrected for small samples (AICc) explicitly penalizes excessive model complexity [31], while cross-validation that withholds an entire mixture avoids placing freeze–thaw nodes from the same mixture in both training and validation sets. Accordingly, the novelty of the present study does not lie in using a Weibull function itself, which has already been applied to freeze–thaw damage [26,27], but in constructing a parsimonious mix-to-Weibull-parameter mapping and evaluating it with AICc, leave-one-mixture-out cross-validation, and non-orthogonal mixtures within the same material system.

Accordingly, this study uses an L_9_(3^3^) orthogonal design to investigate the effects of the total mineral admixture replacement rate, fly ash to slag powder mass ratio, and PVA fiber volume content on apparent morphology, mass loss, and relative dynamic elastic modulus during 200 rapid freeze–thaw cycles. A two-parameter Weibull function is first used to characterize the measured damage evolution and is compared with a classical exponential model in the original damage space. A low-dimensional empirical mapping from mix variables to Weibull characteristic parameters is then constructed under an explicit parameter count constraint and evaluated using AICc, leave-one-mixture-out cross-validation, and three non-orthogonal mixtures from the same material system. The resulting equation is intended for local trend analysis and preliminary screening within the calibrated material system and parameter range, not for unrestricted extrapolation or cross-material service life prediction.

## 2. Experimental Section

### 2.1. Raw Materials

**Figure 1:**
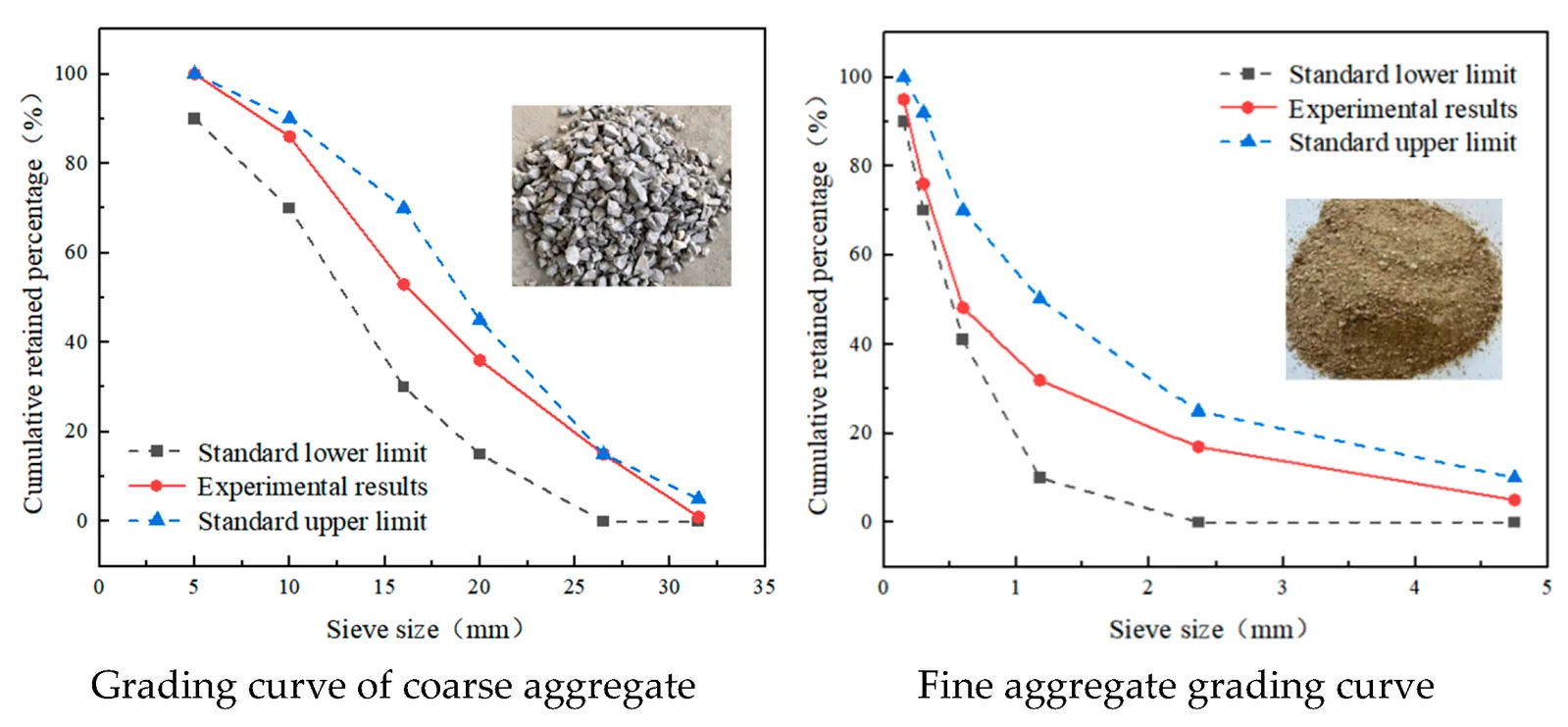
Grading curves of coarse and fine aggregates.

The cement used was P.O 42.5 ordinary Portland cement from Taiyuan Shitou Cement Co., Ltd. (Taiyuan, China); its chemical composition is shown in Table 1. Fly ash and S95 slag powder were both produced by Henan Jinrun New Materials Co., Ltd. (Zhengzhou, China), with specific surface areas of 450 m^2^/kg and 429 m^2^/kg, respectively; their chemical compositions are also listed in Table 1. PVA fibers were produced by Shandong Daitian Engineering Materials Co., Ltd. (Tai’an, China), with a length of 12 mm and a diameter of 5–20 μm; their main performance indicators are shown in Table 2. Both coarse and fine aggregates were sourced locally in Taiyuan; the coarse aggregate consisted of 5–20 mm continuously graded crushed stone, while the fine aggregate was natural river sand. Their physical performance indicators are shown in Table 3. Sieving tests were conducted in accordance with the “Standard for Quality and Testing Methods of Sand and Aggregates for Ordinary Concrete” (JGJ 52-2006) [32], and the gradation curve is shown in Figure 1. The water-reducing agent used was a high-efficiency polycarboxylic acid water-reducing agent from Shanxi Feike New Materials Technology Co., Ltd. (Yuncheng, China), and ordinary tap water was used for mixing.

### 2.2. Mix Design and Testing Methods

The experiments were designed using an L_9_(3^3^) orthogonal array, with the following factors: total replacement rate of the composite admixture (20%, 30%, and 40%); fly ash–slag powder mass ratio (1:1, 1:2, and 2:1); and PVA fiber volume content (0.1%, 0.3%, and 0.5%), with an additional plain concrete control group, resulting in a total of 10 mix designs. The water to binder ratio was fixed at 0.45. The mix design parameters for the PVA fiber–fly ash–slag powder concrete (PVA-FA-SPC) specimens are shown in Table 4.

**Figure 2:**
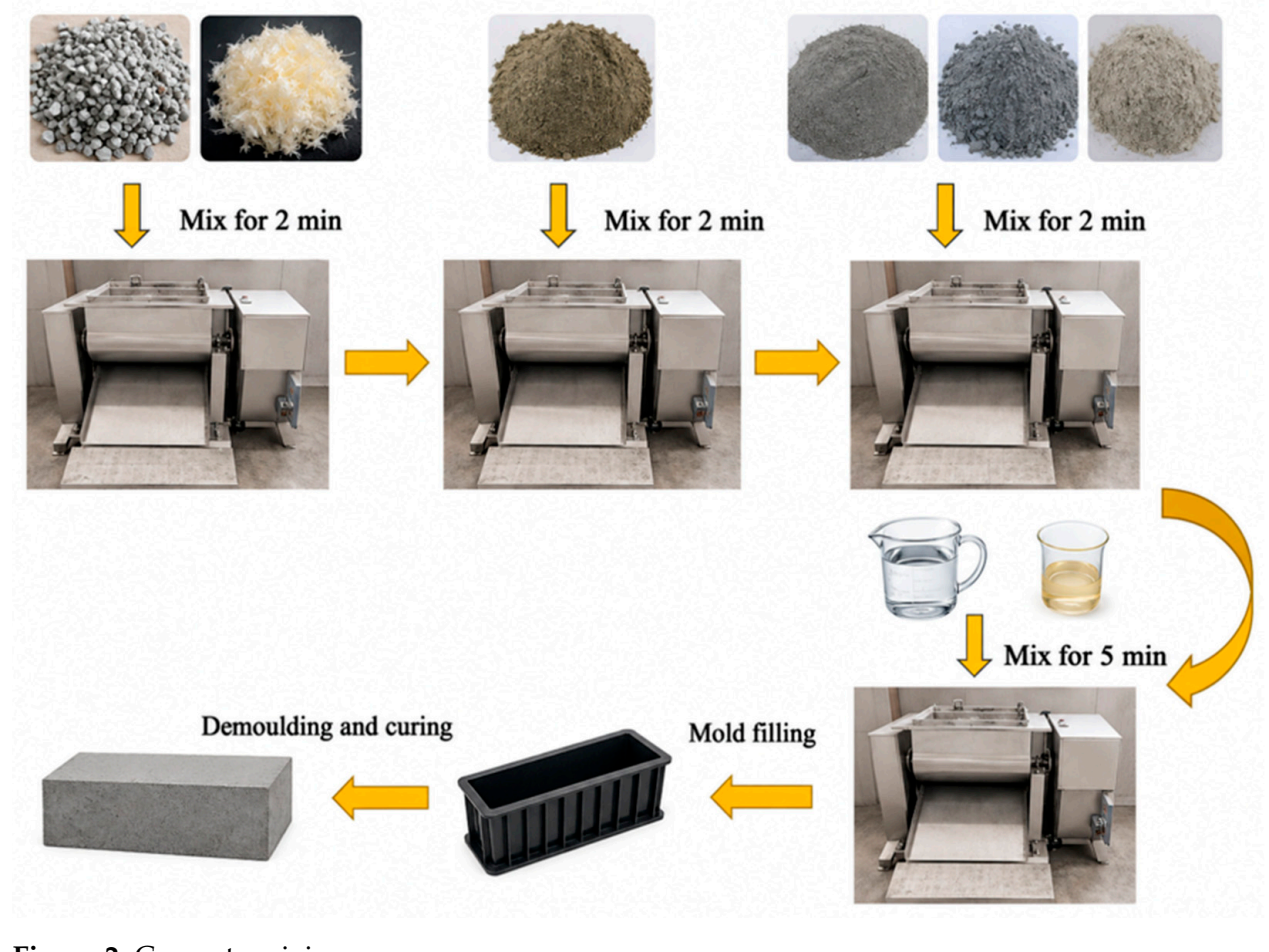
Concrete mixing process.

Prismatic specimens measuring 100 mm × 100 mm × 400 mm were prepared, with three parallel specimens for each mix design. After 28 days of standard curing, rapid freeze–thaw testing was conducted in accordance with GB/T 50082-2009 [33] for a maximum of 200 cycles. The test apparatus comprised a rapid freeze–thaw chamber and a DT-20W dynamic elastic modulus tester operated in the transverse-vibration mode. At each scheduled measurement interval, visible surface water was removed before weighing to obtain a consistent surface-dry condition. The transverse fundamental resonance frequency and mass were measured at 0 cycles and after every 25 cycles. The relative dynamic elastic modulus was calculated directly from the squared ratio of the transverse fundamental resonance frequencies, as defined in Equation (2). Freeze–thaw resistance was evaluated using the mass loss rate and relative dynamic elastic modulus. The concrete mixing process is shown in Figure 2.

The retained freeze–thaw dataset consists of group mean responses at each cycle node. These group means were used directly for damage curve fitting and subsequent model development; individual specimen-level measurements were not available and were therefore neither reconstructed nor treated as independent observations.The corresponding group-level cycle-node dataset is provided in Supplementary File S1.

## 3. Analysis of Test Results

### 3.1. Evolution of Apparent Damage

**Figure 3:**
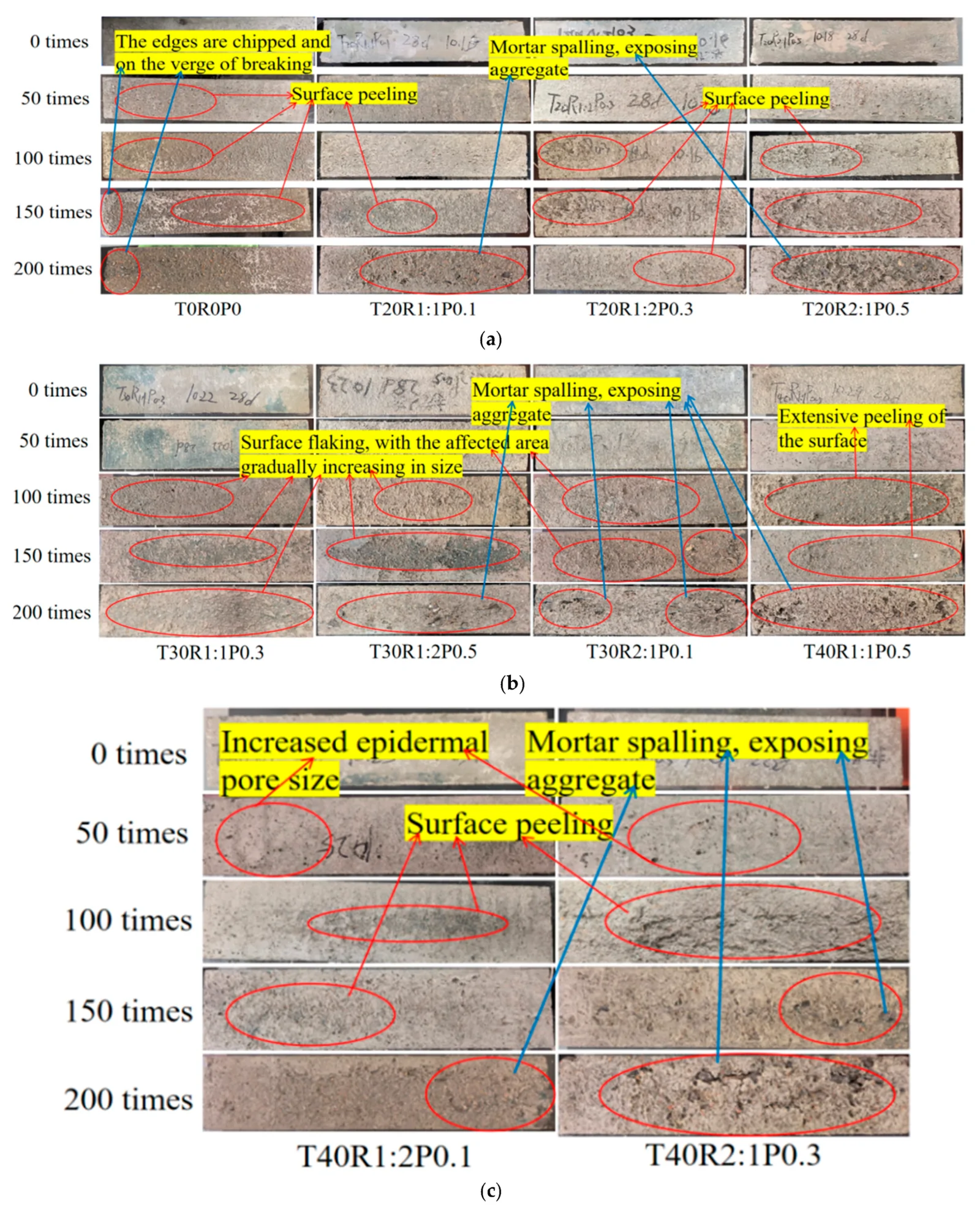
Changes in the appearance of PVA-FA-SPC during freeze–thaw cycling: (**a**) T0R0P0, T20R1:1P0.1, T20R1:2P0.3, and T20R2:1P0.5; (**b**) T30R1:1P0.3, T30R1:2P0.5, T30R2:1P0.1, and T40R1:1P0.5; and (**c**) T40R1:2P0.1 and T40R2:1P0.3. Red circles highlight representative damaged regions, blue leader lines connect the labels to the corresponding features, and yellow text boxes describe the observed damage types.

Figure 3 shows the apparent damage patterns of PVA-FA-SPC after different numbers of freeze–thaw cycles. During the freeze–thaw cycles, the apparent damage to the concrete gradually worsened: after 0 cycles, the surface was smooth; after 50 cycles, slight spalling occurred; after 100 cycles, mortar spalled off and a small amount of aggregate was exposed; after 150 cycles, the edges and corners were fractured and the aggregate was significantly exposed; after 200 cycles, the control group exhibited extensive mortar spalling and spalling at the edges and corners, while the test groups showed significantly less damage. Among them, the T20R1:2P0.3 group exhibited the best visual integrity, with only minor localized spalling. Overall, the results indicate that as the replacement rate increases and the fiber content deviates from 0.3%, the severity of visual damage increases.

### 3.2. Mass Loss Rate

**Figure 4:**
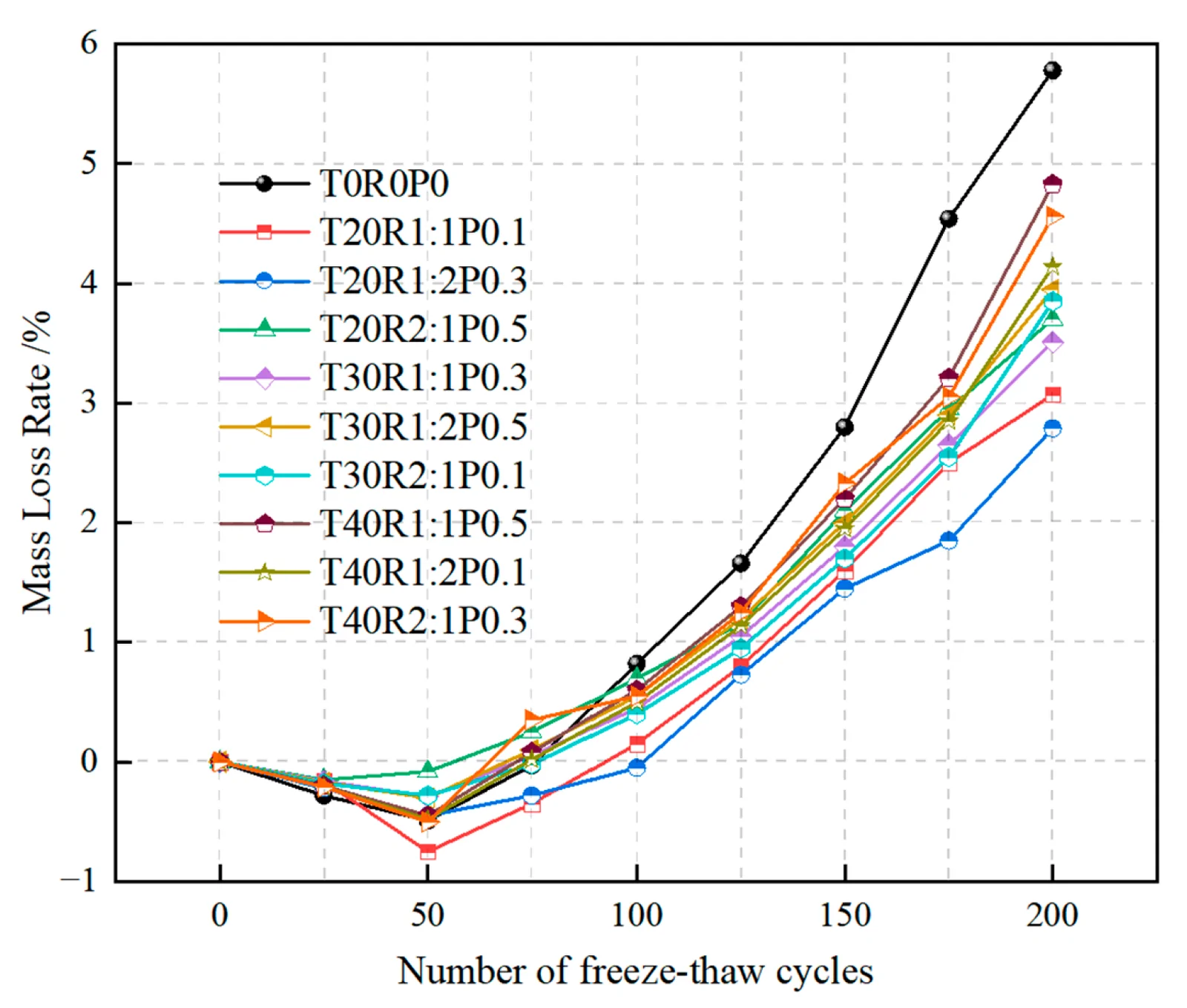
Relationship between mass loss and number of freeze–thaw cycles.

The mass loss rate of the test specimen is calculated using Equation (1); the corresponding curve illustrating this relationship is shown in Figure 4:

(1) $$\begin{array}{c} \delta_{n}\;=\;\frac{m_{0}\;-\;m_{n}}{m_{0}}\;\times \;100\% \end{array}$$

In Equation (1), δ_n_ is the mass loss rate after n freeze–thaw cycles (%), while m_0_ and m_n_ are the specimen masses before freeze–thaw exposure and after n cycles, respectively (kg). Mass was determined only after the prescribed surface-conditioning step so that free surface water was not included in the weighing result.

The mass loss curves for all mixtures are shown in Figure 4. A common pattern of initial mass gain followed by mass loss was observed. Because the specimens were weighed under a consistent surface-dry protocol, the negative mass loss values in the early stage are interpreted as net water uptake into accessible pores rather than residual free water on the specimen surface. After approximately 50 cycles, cumulative internal damage and surface spalling became dominant, and the mass loss rate turned positive and increased rapidly.

After 200 cycles, the mass loss rate of the reference group reached 5.78%, exceeding the 5% limit specified in the standard; the loss rates of all test groups were less than 5%, meeting the standard requirements. Among them, the T20R1:2P0.3 group had the lowest loss rate and demonstrated the best freeze–thaw resistance.

### 3.3. Relative Dynamic Modulus of Elasticity

**Figure 5:**
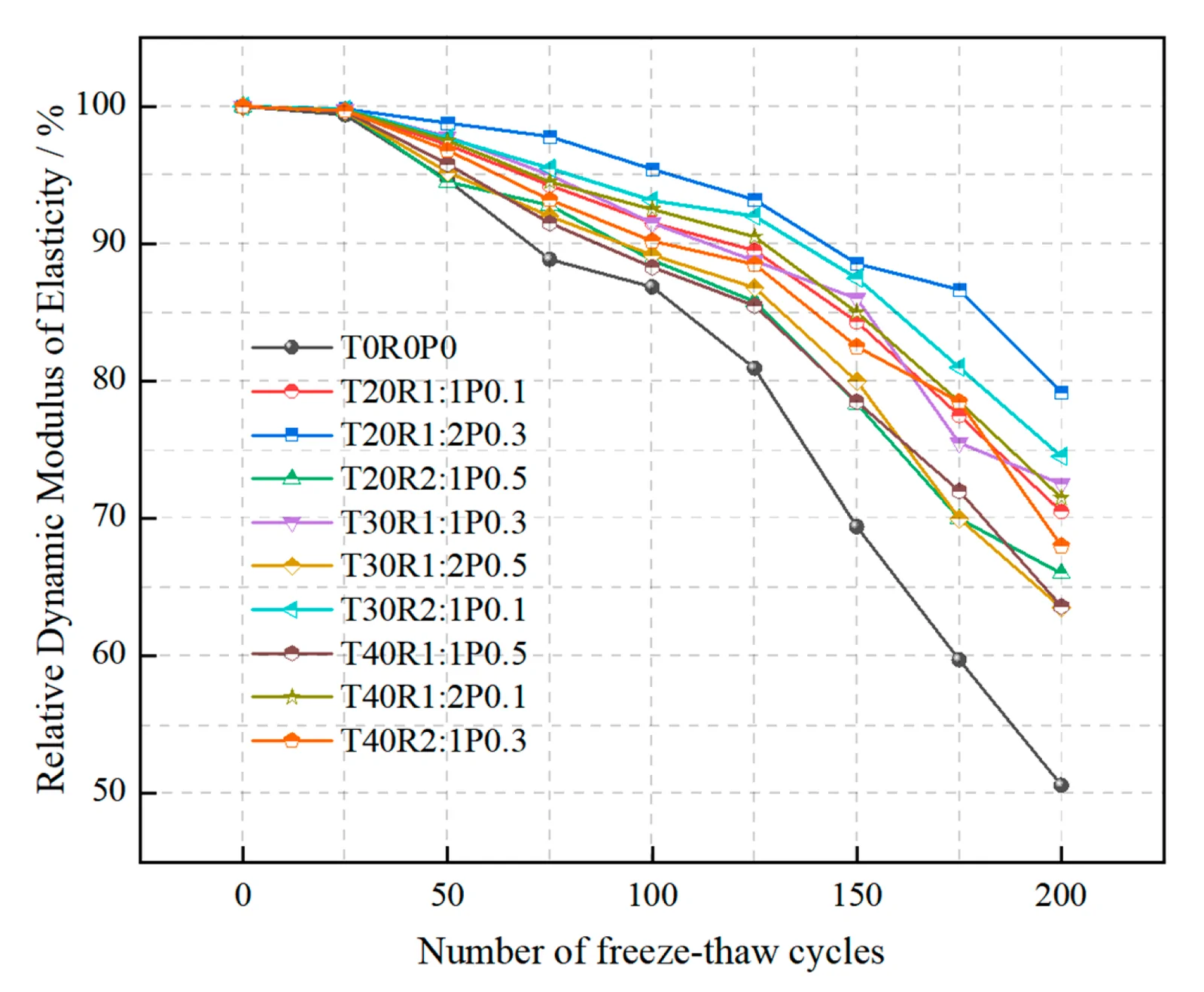
Relationship between relative dynamic elastic modulus and number of freeze–thaw cycles.

The relative dynamic elastic modulus was calculated from the transverse fundamental resonance frequency measured using the DT-20W dynamic elastic modulus tester in accordance with GB/T 50082-2009 [33], as shown in Equation (2). The resulting evolution curves are presented in Figure 5.

(2) $$\begin{array}{c} p_{n}={\left(\frac{f_{n}}{f_{0}}\right)}^{2}\times 100\% \end{array}$$

In Equation (2), *p_n_* is the relative dynamic elastic modulus after *n* freeze–thaw cycles (%), while *f*_0_ and *f_n_* are the transverse fundamental resonance frequencies before freeze–thaw cycling and after *n* cycles, respectively (Hz). Thus, the relative dynamic elastic modulus is calculated directly as *p_n_* = (*f_n_*/*f*_0_)^2^ × 100%.

The relative dynamic elastic modulus is a key indicator for evaluating freeze–thaw damage; the evolution curves for each group are shown in Figure 5. As the number of freeze–thaw cycles increases, the relative dynamic elastic modulus continues to decrease; the decline was gradual between 0 and 50 cycles, with minimal differences among the groups; after 50 cycles, damage accelerated, and the differences between groups gradually widened.

After 200 cycles, the relative dynamic elastic modulus of the reference group dropped to 50.61%, below the 60% failure threshold; all test groups remained above 63%, with the T20R1:2P0.3 group reaching 79.18%, the best performance among all groups. The overall trend was consistent with the mass loss rate: as the total substitution rate increased and the fiber content deviated from 0.3%, the loss of the dynamic elastic modulus became more pronounced.

### 3.4. Range Analysis and Exploratory Main Effect Decomposition of the Orthogonal Experiment

The relative dynamic elastic modulus after 200 freeze–thaw cycles was selected as the response variable to evaluate the effects of the total mineral admixture replacement rate (factor A), the fly ash to slag powder mass ratio (factor B), and the PVA fiber volume content (factor C). The factor levels and the measured mean responses of the L_9_(3^3^) orthogonal experiment are presented in Table 5 and Table 6, respectively. The plain-concrete group T0R0P0 was used only as a reference and was not included in the orthogonal effect analysis.

Range analysis was first employed to compare the relative influence of the three factors. For each factor, K_i_ denotes the sum of the response values corresponding to level i, k_i_ = K_i_/3 represents the corresponding mean response, and the range R is calculated as the difference between the maximum and minimum k_i_ values. A larger R indicates a stronger variation in the relative dynamic elastic modulus over the investigated factor levels.

**Figure 6:**
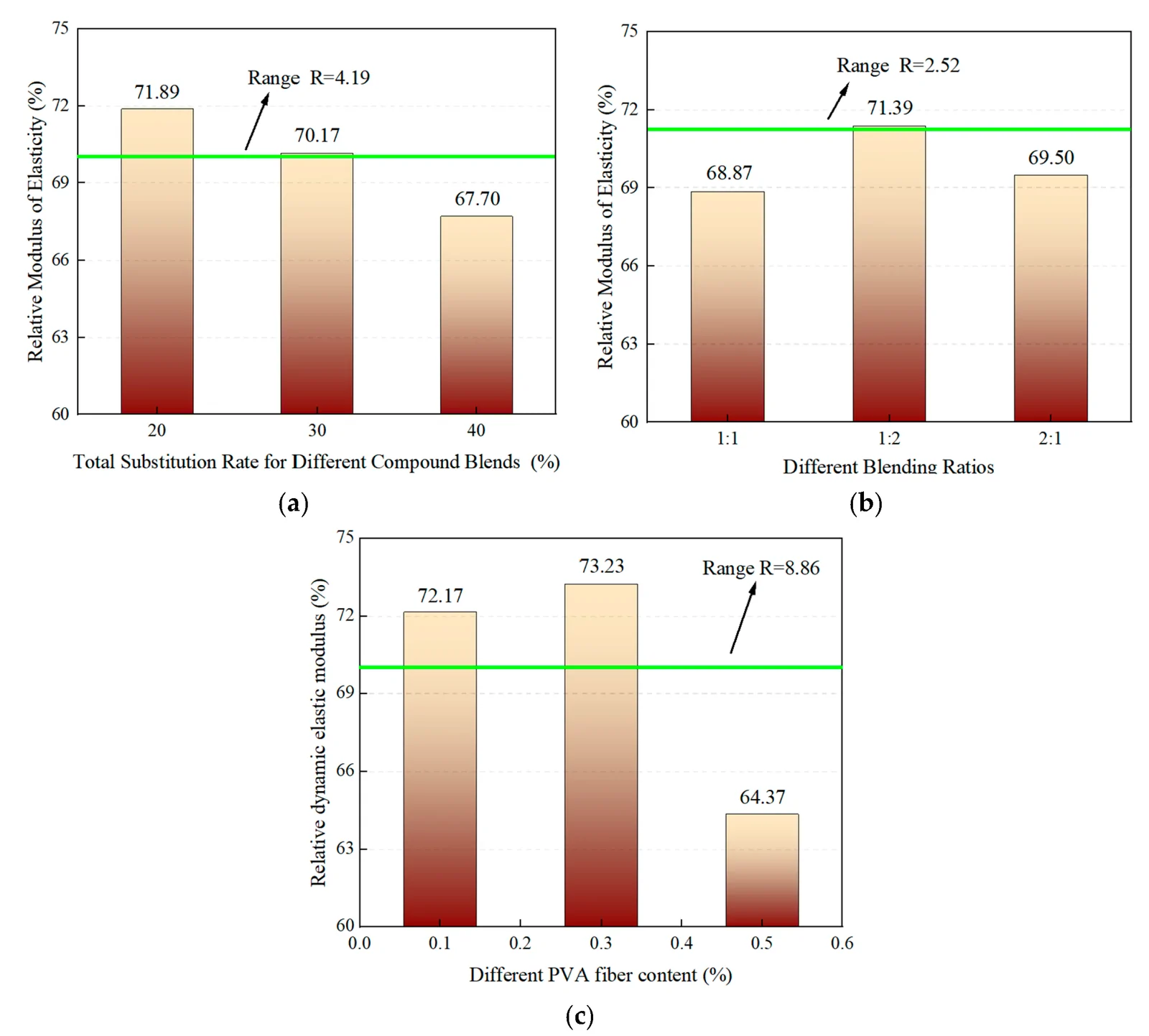
Main-effect trends for the relative dynamic elastic modulus after 200 freeze–thaw cycles: (**a**) total mineral-admixture replacement rate; (**b**) fly ash-to-slag powder mass ratio; (**c**) PVA fiber volume content.

For factor A, the mean relative dynamic elastic moduli at replacement rates of 20%, 30%, and 40% were 71.89%, 70.17%, and 67.70%, respectively, giving a range of R_A = 4.19. For factor B, the corresponding mean values at fly ash to slag powder ratios of 1:1, 1:2, and 2:1 were 68.87%, 71.39%, and 69.50%, respectively, resulting in R_B = 2.52. For factor C, the mean values at PVA fiber contents of 0.1%, 0.3%, and 0.5% were 72.17%, 73.23%, and 64.37%, respectively, and the corresponding range was R_C = 8.86. Therefore, the factor influence determined from the range analysis follows the order C > A > B, i.e., PVA fiber volume content > total mineral admixture replacement rate > fly ash to slag powder mass ratio. The main effect trends are illustrated in Figure 6.

The results indicate that increasing the total mineral admixture replacement rate from 20% to 40% generally reduced the relative dynamic elastic modulus after 200 freeze–thaw cycles. For the fly ash to slag powder ratio, the 1:2 level produced the highest mean response within the investigated range. The effect of PVA fiber content was more pronounced: increasing the fiber content from 0.1% to 0.3% slightly improved the mean relative dynamic elastic modulus, whereas a further increase to 0.5% resulted in a marked reduction. This behavior suggests that a moderate PVA fiber content can improve resistance to freeze-thaw-induced stiffness degradation, whereas excessive fiber incorporation may offset this benefit, potentially because of reduced dispersion uniformity and additional interfacial or entrapped air defects.

Because the analysis is based on one retained mean response for each orthogonal mixture and specimen-level replicate data are unavailable for estimating pure experimental error, the statistical decomposition in this section is treated as exploratory rather than as confirmatory significance testing. In particular, the residual term of an additive main effect model cannot be interpreted as an independent estimate of pure experimental error because it may contain contributions from unmodeled interactions, nonlinear effects, and experimental variability. Therefore, F-tests and *p*-values are not used as the primary basis for determining factor significance. Instead, an exploratory sum-of-squares decomposition was used to provide an additional descriptive measure of the relative magnitude of the main effects, as summarized in Table 7.

As shown in Table 7, the sums of squares associated with factors A, B, and C were 26.65, 10.37, and 140.46, respectively, accounting for 12.31%, 4.79%, and 64.90% of the total sum of squares. The remaining residual component accounted for 17.99%. These proportions are used only to describe the relative magnitudes of the effects within the present L_9_ experimental dataset and should not be interpreted as confirmatory estimates of statistical significance.

Both the range analysis and the exploratory sum-of-squares decomposition consistently identify PVA fiber content as the factor associated with the largest variation in the relative dynamic elastic modulus, followed by the total mineral admixture replacement rate and then the fly ash to slag powder ratio. Based on the factor-level means, the preferable level combination within the investigated range is A1B2C2, corresponding to a total mineral admixture replacement rate of 20%, a fly ash to slag powder mass ratio of 1:2, and a PVA fiber volume content of 0.3%. This combination is represented by specimen T20R1:2P0.3, which exhibited a measured relative dynamic elastic modulus of 79.18% after 200 freeze–thaw cycles, the highest value among the tested composite concrete mixtures. Accordingly, this combination is regarded as the experimentally best-performing mixture within the discrete factor levels examined in this study, rather than as a universally optimized mix proportion.

## 4. Development and Validation of a Freeze–Thaw Damage Model

Damage to the relative dynamic elastic modulus during freeze–thaw cycles typically manifests as a nonlinear process characterized by slow accumulation in the early stages and accelerated progression in the later stages. The Weibull function, with its small number of parameters and adjustable curve shape, is well suited for phenomenological descriptions of this type of monotonic damage process [34,35]. This paper first establishes a two-parameter Weibull-type damage model for each mix proportion and compares it with the classical exponential model; subsequently, a low-dimensional empirical mapping of mix proportion parameters is constructed using the Weibull characteristic parameters as the response.

### 4.1. Weibull Model for the Evolution of Freeze–Thaw Damage

Based on the two-parameter Weibull distribution, let *ln* be the probability density function after n freeze–thaw cycles, as shown in Equation (3):

(3) $$\begin{array}{c} l\left(n\right)\;=\;\frac{\alpha }{\beta }{\left(\frac{n}{\beta }\right)}^{\alpha -1}exp\left[-{\left(\frac{n}{\beta }\right)}^{\alpha }\right] \end{array}$$

In the equation, α is the shape parameter describing the rate and concentration of damage development; a larger α corresponds to a steeper transition over a narrower cycle interval, whereas a smaller α indicates a more gradual evolution. β is the Weibull scale parameter characterizing the horizontal cycle scale of the fitted damage curve. A larger β shifts the fitted damage evolution toward a larger number of cycles; β itself should not be interpreted as a measured failure cycle count or service life. n is the number of freeze–thaw cycles.

Integrating the Weibull probability density function in Equation (3) yields the cumulative distribution function L_n_, as shown in Equation (4):

(4) $$\begin{array}{c} L\left(n\right)\;=\;1\;-\;exp\left[-{\left(\frac{n}{\beta }\right)}^{\alpha }\right] \end{array}$$

This paper defines a damage variable based on the relative dynamic elastic modulus and establishes a phenomenological correspondence between it and the Weibull cumulative distribution function, taking advantage of the mathematical characteristics that the damage degree shares the same initial boundary, monotonicity, and range of values as the Weibull cumulative distribution function. This approach is used to describe the shape of experimental curves and does not imply that the damage degree is, in a strict statistical sense, equivalent to the failure probability obtained from a large number of independent failure samples.

Accordingly, the freeze–thaw damage degree can be expressed as Equation (5):

(5) $$\begin{array}{c} D\left(n\right)\;=\;1\;-\;exp\left[-{\left(\frac{n}{\beta }\right)}^{\alpha }\right] \end{array}$$

For ease of calculation, Equation (5) is rearranged and then taken to the natural logarithm, yielding Equation (6):

(6) $$\begin{array}{c} ln\left(\ln\frac{1}{1\;-\;D\left(n\right)}\right)\;=\;\alpha ln\left(n\right)\;-\;\alpha ln\beta \end{array}$$

Let *y*$\;=\;\ln\left(\ln\frac{1}{1-D\left(n\right)}\right)$, X = ln(n), a = α, and *b* = −αlnβ. Then, the linear regression equation is given by Equation (7):

(7) $$\begin{array}{c} y\;=\;ax\;+\;b \end{array}$$

### 4.2. Weibull Parameter Fitting and Curve Characterization

A linear regression was performed after Weibull linearization using the measured mean damage nodes at 25, 50, 75, 100, 125, 150, 175, and 200 cycles. Table 8 reports the linearized regression coefficients a and b, the coefficient of determination in the transformed ln–ln space (R^2^_ln–ln), and the coefficient of determination (R^2^_D) and root mean square error (RMSE_D) evaluated in the original damage space after back-transformation.

The shape parameter α and scale parameter β were calculated from a and b to obtain the Weibull-type damage curve for each mixture; the fitted parameters and corresponding mixture-specific Weibull equations are summarized in Table 9. The R^2^_ln–ln values in Table 8 (0.939–0.995) describe only the linearized transformed space. The separately reported R^2^_D and RMSE_D values quantify agreement with the measured nodes in the original damage space. These metrics support the descriptive adequacy of the Weibull curves for the measured nodes but do not establish predictive capability for untested material systems.

**Figure 7:**
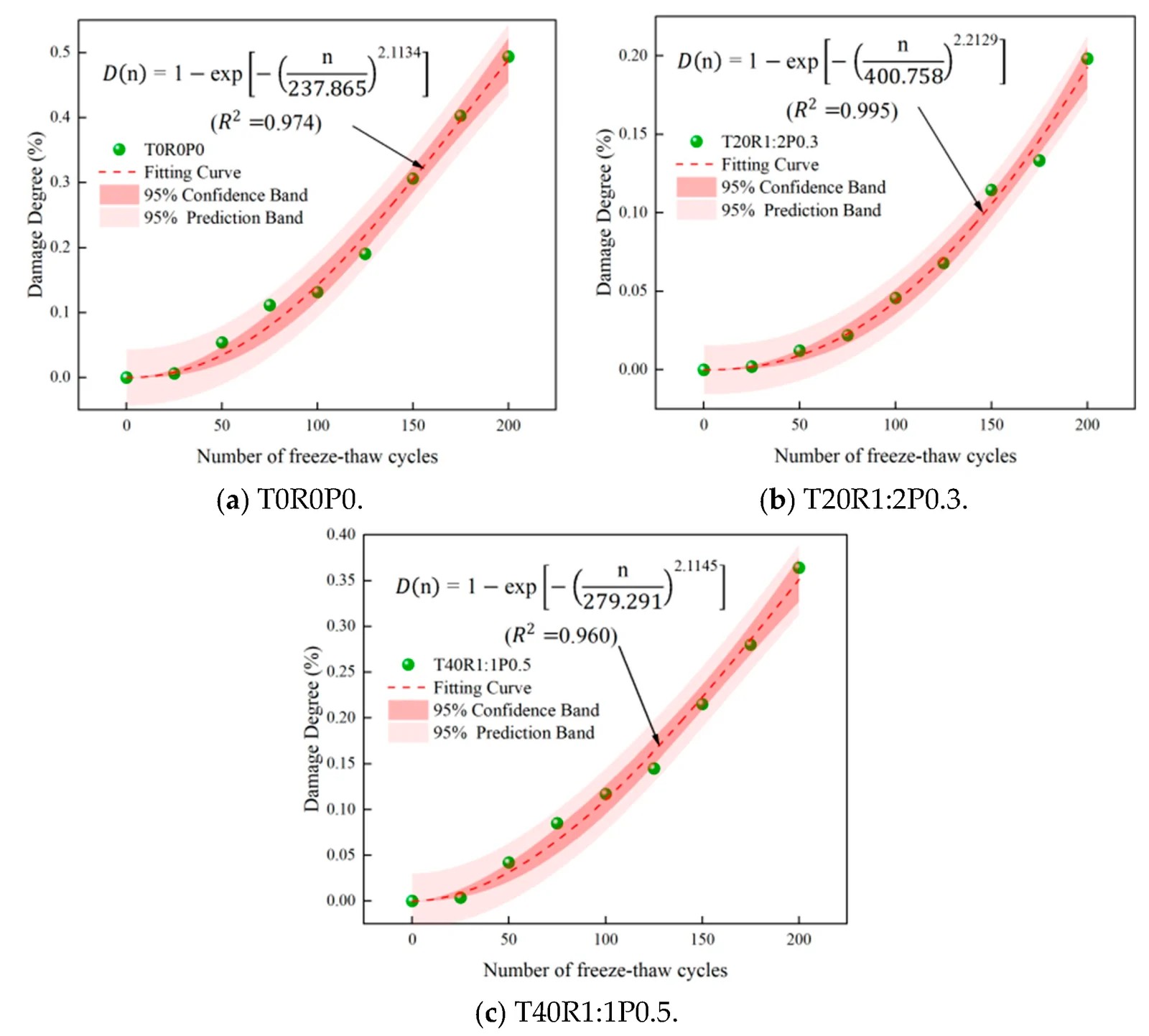
Comparison of damage levels calculated using the freeze–thaw damage model with measured values.

Data from three groups—the reference group, the group with the best test results, and the group with typical high damage—were selected, and the Weibull-fitted curves were compared with the measured damage levels at each freeze–thaw cycle node. The results are shown in Figure 7. The curves for all three groups reflect a damage evolution pattern characterized by a slow initial phase followed by an accelerated later phase, and they generally agree with the measured data from the nodes.

### 4.3. Comparison of In-Sample Fitting Accuracy

**Figure 8:**
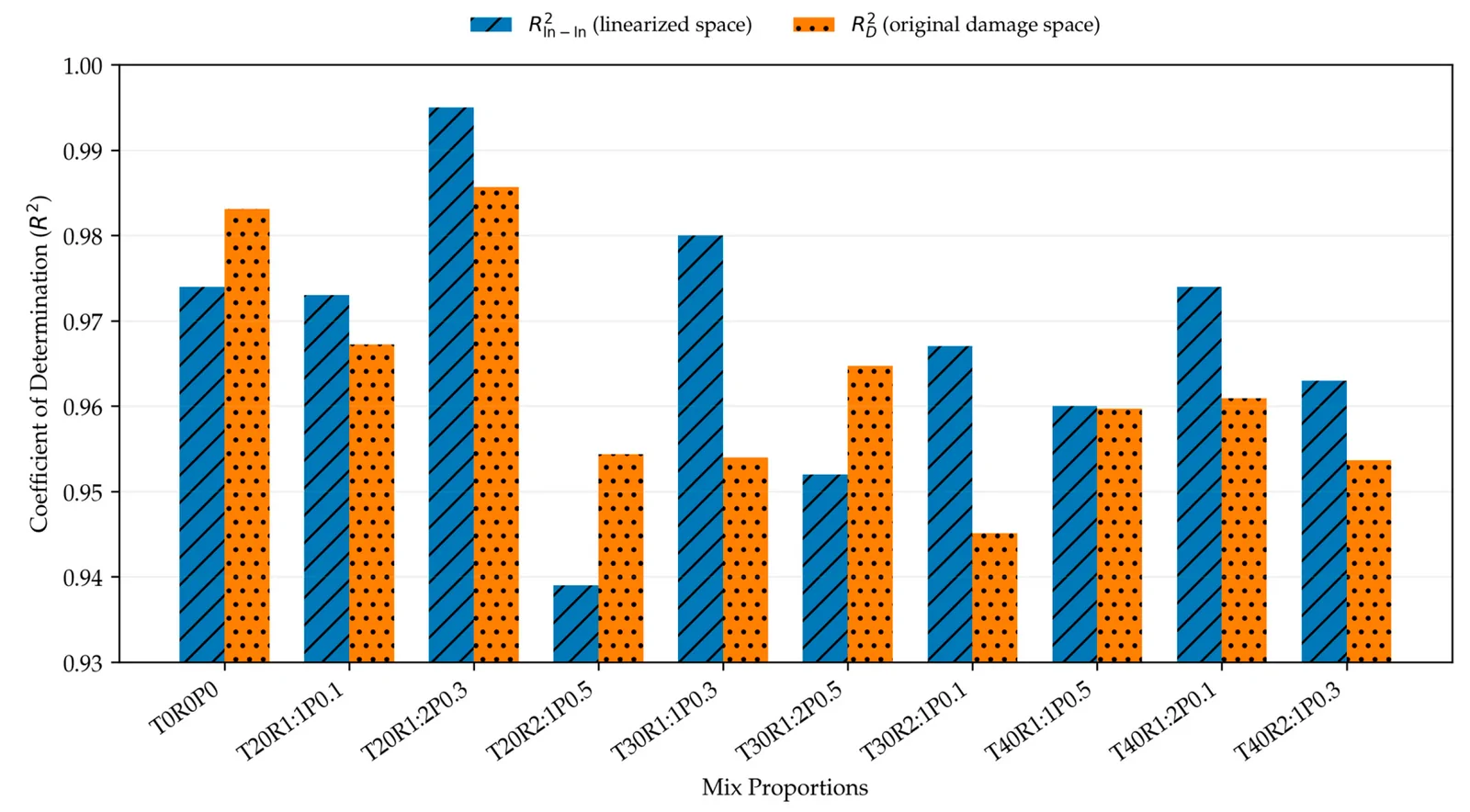
Comparison of Weibull R^2^ in the linearized and original damage spaces.

A classical exponential damage model was used as a benchmark (Equation (8)). Cross-model comparison emphasizes error metrics calculated in the original damage space, namely root mean square error (RMSE) and mean absolute percentage error (MAPE), as defined in Equations (9) and (10). For the Weibull model, Table 8 and Figure 8 separately distinguish the transformed space R^2^_ln–ln from the original damage space R^2^_D so that these coefficients are not conflated:

(8) $$\begin{array}{c} D\left(n\right)\;=\;1\;-\;exp(-kn) \end{array}$$

In the equation, D(n)—the degree of freeze–thaw damage after n freeze–thaw cycles; k—the damage rate constant, obtained by linear fitting of experimental data using the least squares method; and n—the number of freeze–thaw cycles.

(9) $$\begin{array}{c} RMSE=\;\sqrt{\frac{1}{N}\sum_{i=1}^{N}{\left(D_{\mathrm{Field}\;\mathrm{Test},i}\;-\;D_{\mathrm{Forecast},i}\right)}^{2}} \end{array}$$

(10) $$\begin{array}{c} MAPE=\frac{1}{N}\sum_{i=1}^{N}\left|\frac{D_{\mathrm{Field}\;\mathrm{Test},i}\;-\;D_{\mathrm{Forecast},i}}{D_{\mathrm{Field}\;\mathrm{Test},i}}\right| \end{array}$$

In Equations (9) and (10), N is the number of freeze–thaw nodes used for each mixture (N = 8); D_i_ is the measured mean damage degree at node I; and D_i_ is the corresponding fitted value. Smaller RMSE and MAPE values indicate a smaller discrepancy in the original damage space.

Because early-stage measured damage values can be close to zero, MAPE can magnify small absolute deviations. Accordingly, RMSE in the original damage space is treated as the primary fitting error metric, with MAPE used as supplementary information.

Figure 8 compares the coefficient of determination from the Weibull linearized regression (R^2^_ln–ln) with the coefficient calculated directly in the original damage space (R^2^_D) for each mixture. The two statistics refer to different response spaces and are therefore reported separately rather than used as a cross-model accuracy comparison.

**Figure 9:**
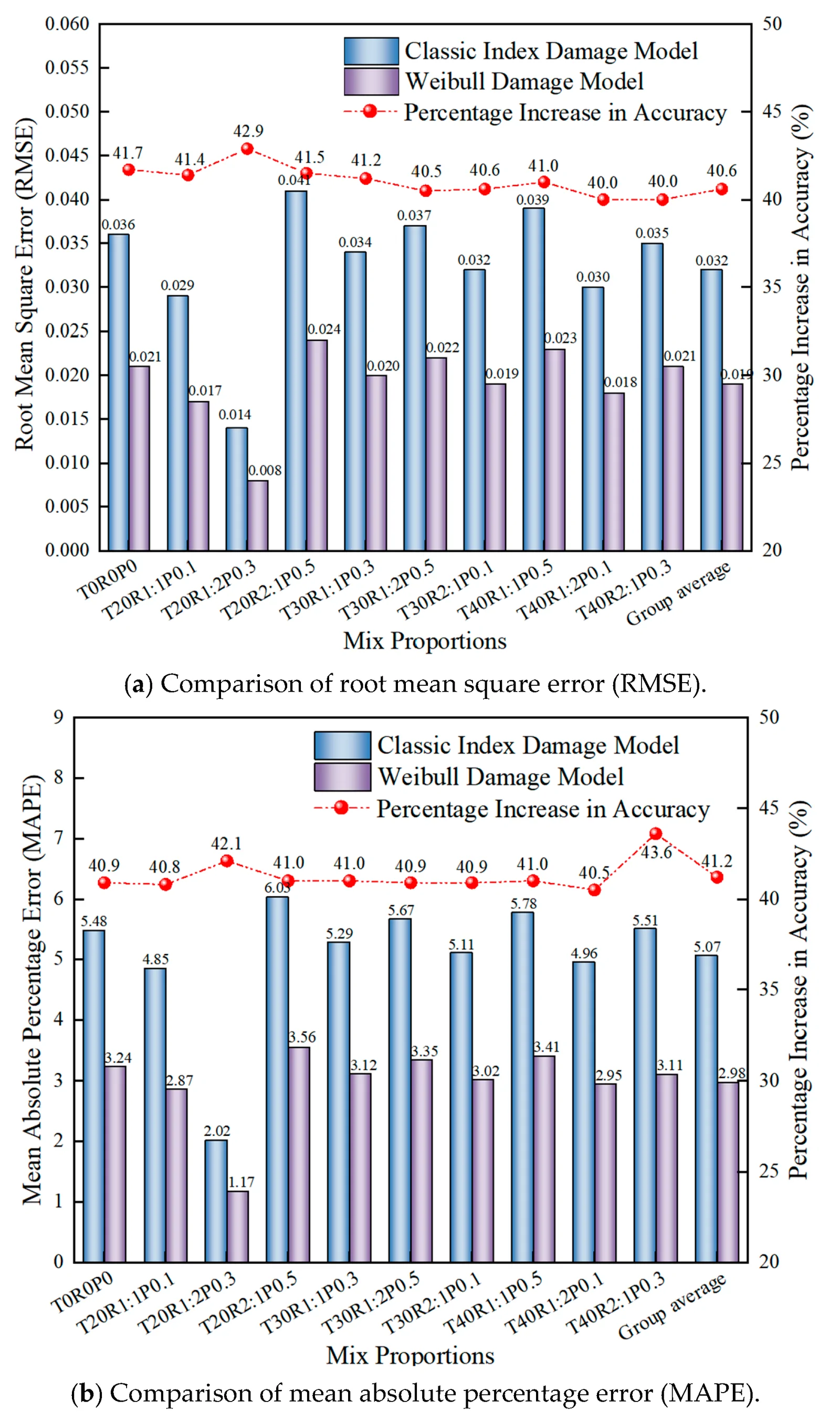
Comparison of in-sample fitting errors for the classical exponential and Weibull models.

The original space R^2^_D values confirm that the Weibull curves provide a generally close in-sample description of the measured damage nodes, while the differences from R^2^_ln–ln illustrate why transformed space goodness of fit should not be interpreted as an original space metric. The direct comparison between the Weibull and classical exponential models is therefore based on the original damage space RMSE and MAPE values shown in Figure 9.

The quantitative comparison of the original damage space fitting errors for the two models is shown in Figure 9.

As shown in Figure 9, the Weibull model gives lower original space RMSE and MAPE values than the classical exponential model for all mixtures. The average RMSE decreases from 0.032 to 0.019, and the average MAPE decreases from 5.07% to 2.98%; for T20R1:2P0.3, the Weibull MAPE is 1.17%. These results demonstrate improved in-sample description of the measured nonlinear damage curves. They should not be interpreted as evidence of out-of-sample predictive accuracy, which is evaluated separately through LOMO-CV and the non-orthogonal validation mixtures.

The individual-mixture Weibull fits are post hoc descriptions because α and β are estimated using the nodes of the same mixture. To obtain a mapping that can be evaluated on mixtures omitted from parameter estimation, the next section links mix variables to the characteristic parameters under a restricted model structure and assesses its local out-of-sample stability.

### 4.4. Low-Dimensional Empirical Mapping of Mix Proportion Parameters and Weibull Parameters

#### 4.4.1. Modeling Approach and Variable Encoding

Only nine independent orthogonal mixtures were available for empirical parameter mapping. Introducing numerous linear, quadratic, and interaction terms under this small-sample condition would reduce parameter identifiability and inflate in-sample fit. Therefore, a small, predefined set of candidate equations was evaluated jointly using AICc and leave-one-mixture-out cross-validation (LOMO-CV), without reselecting variables within individual folds. AICc balances fit against model complexity, while LOMO-CV evaluates interpolation to an omitted mixture within the investigated material system and factor ranges; it is not equivalent to validation across different material sources, water to binder ratios, or production batches.

The total replacement rate of mineral admixtures (T), the mass fraction of slag powder relative to the total mineral admixtures (R), and the volumetric dosage of PVA fibers (P) are encoded into dimensionless variables as follows:

(11) $$\begin{array}{c} x_{T}\;=\;(T\;-\;30)/10;\;x_{R}\;=\;(R\;-\;0.5)/0.1667;\;x_{P}\;=\;(P\;-\;0.3)/0.2 \end{array}$$

Specifically, the range of T is 20–40%, the range of R is 0.333–0.667, and the range of P is 0.1–0.5%. After encoding, the values of each variable fall within the range of approximately –1 to 1, which helps reduce dimensional differences and improve the numerical stability of parameter estimates.

The shape parameter α for the nine groups of composite concrete ranges from 2.0692 to 2.2367, with a mean of 2.1403 and a coefficient of variation of 2.56%. Compared to the scale parameter β, α exhibits less intergroup variation; given the current sample size, further fitting of the relationship between α and multiple factors lacks stability. Therefore, α is set as the common shape parameter:

(12) $$\begin{array}{c} \alpha =\alpha 0=2.1403 \end{array}$$

Since the scale parameter β is positive, a logarithmic link function is used to establish an empirical relationship between β and the mix design parameters. Based on the trends observed in the main effects of the orthogonal experiments and the simplicity of the parameters, the following three candidate models are predefined:

(13) $$\begin{array}{c} M1:\ln\beta \;={\;b}_{0}\;+{\;b}_{1}x_{T\;}+\;b_{2}x_{R}\;+\;b_{3}x_{P} \end{array}$$

(14) $$\begin{array}{c} \;\;\;\;\;\;\;\;\;\;\;M2:\ln\beta \;={\;b}_{0}+b_{1}x_{T}+{\;b}_{2}x_{R}+b_{3}x_{P}+b_{4}x_{P^{2}} \end{array}$$

(15) $$\begin{array}{c} M3:\ln\beta =\;b_{0}+b_{1}x_{T}+b_{2}x_{P\;}+b_{3}x_{P^{2}} \end{array}$$

The three candidate models maintain a fixed variable structure in each fold of the cross-validation and do not reselect variables across different folds. During cross-validation, the complete freeze–thaw data for one mix design is removed in each iteration; the equations are then re-estimated using the remaining eight mix designs, and a prediction is made for the removed mix design. This approach prevents data from the same mix design from being included in both the training set and the validation set simultaneously.

#### 4.4.2. Evaluation of Candidate Models and Final Scale Parameter Equations

**Figure 10:**
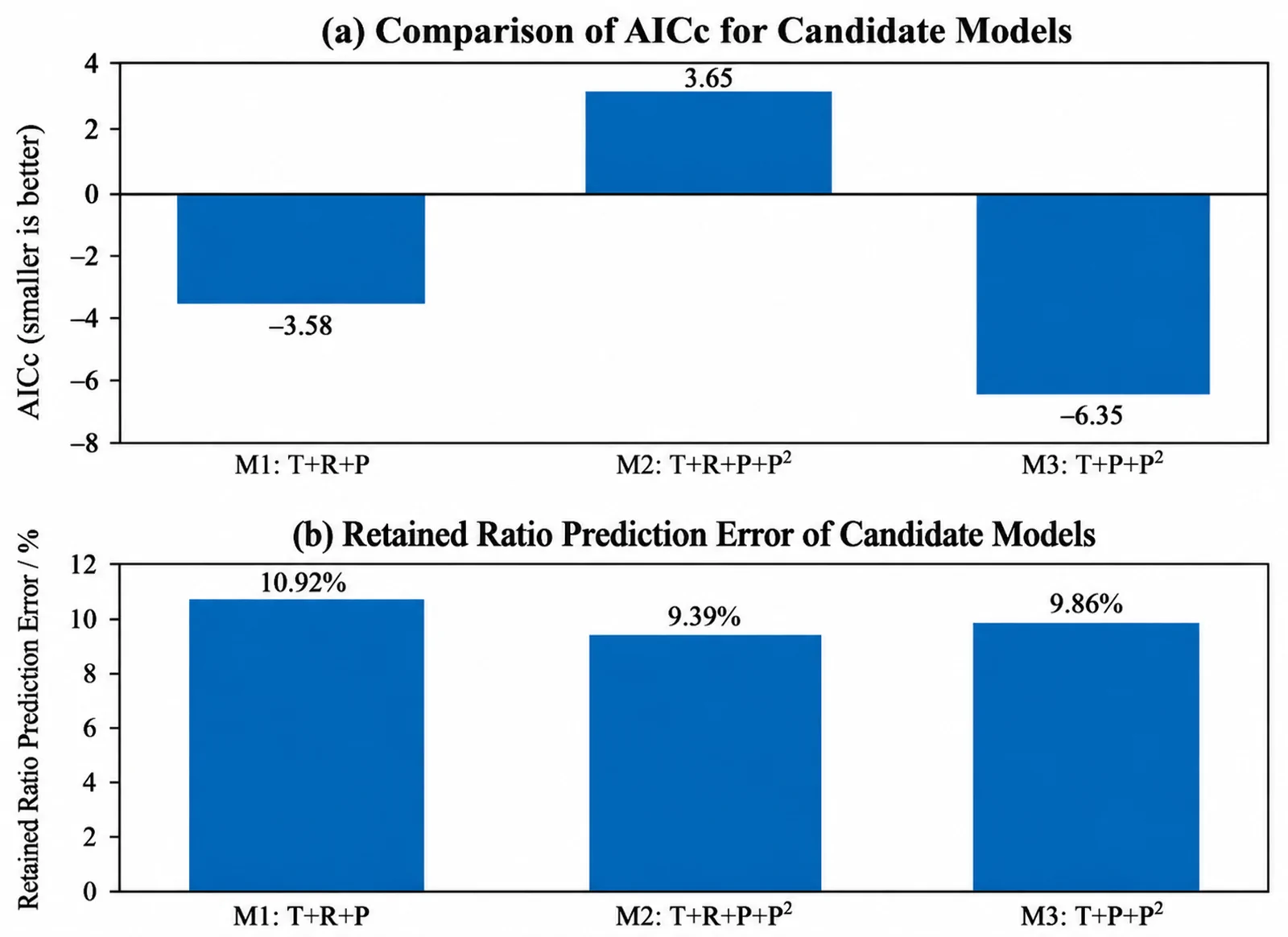
AICc and leave-one-mixture-out cross-validation error of candidate low-dimensional models.

The evaluation results for the candidate models are shown in Figure 10. The adjusted R^2^ values for M1, M2, and M3 were 0.296, 0.483, and 0.483, respectively; their AICc values were −3.58, 3.65, and −6.35, respectively; and the LOMO-CV MAPE for β was 10.92%, 9.39%, and 9.86%, respectively. Model M2 had the smallest cross-validation error, but adding the slag powder proportion term increased the model’s complexity, resulting in a significant increase in AICc. Model M3 reduced the number of parameters by one while retaining the nonlinear term for PVA fiber content; its adjusted R^2^ was essentially the same as that of M2, it had the lowest AICc, and its cross-validation error was only 0.47 percentage points higher than that of M2. Considering model simplicity, parameter stability, and out-of-sample error, M3 was selected as the final scale parameter equation.

The fact that the fly ash–slag composition variable R was not retained in the final equation does not imply that R has no effect on freeze–thaw resistance. Rather, with the present nine mixtures and the imposed complexity limit, the inclusion of R did not yield a sufficiently consistent reduction in out-of-sample error to justify the additional parameter. The influence of the fly ash to slag powder ratio should therefore still be interpreted from the orthogonal experiment in Section 3.4., and the final low-dimensional equation must not be used to optimize R independently.

The final scale parameter equation is as follows:

(16) $$\begin{array}{c} \ln\beta \;=\;5.8306\;-\;0.03822x_{T}-0.08684x_{P}-0.09932x_{P^{2}} \end{array}$$

That is:

(17) $$\begin{array}{c} \beta =\;exp(5.8306\;-\;0.03822x_{T}-0.08684x_{P}-0.09932x_{P^{2}}) \end{array}$$

**Figure 11:**
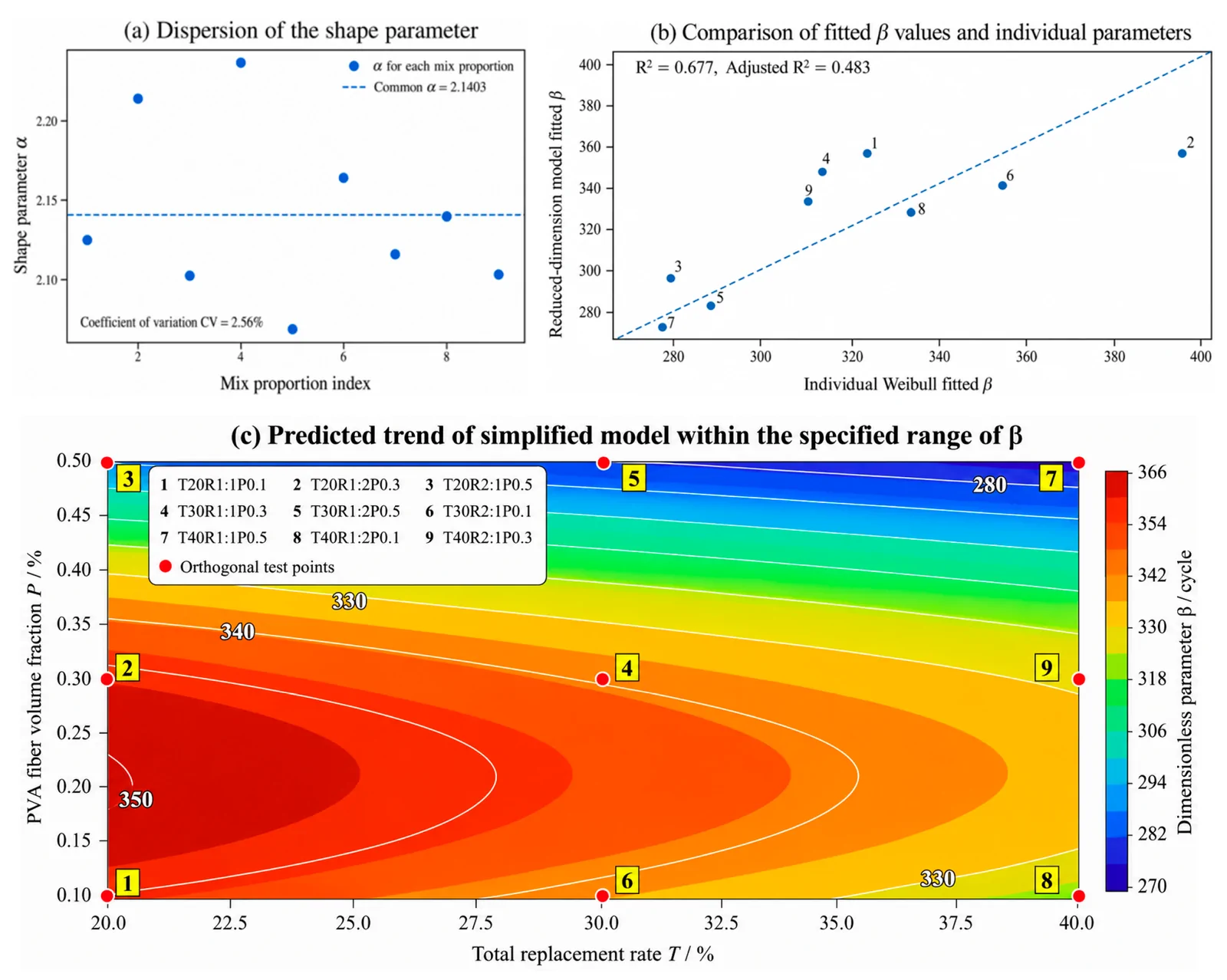
Common shape parameter, scale parameter mapping, and local trends within the calibrated domain.

The final model includes four regression coefficients in the scale parameter equation and one common shape parameter, for a total of five parameters to be estimated. For the nine modeled mix proportions, the R^2^ of the β equation was 0.677, and the adjusted R^2^ was 0.483. Figure 11a shows the dispersion of the shape parameter α; Figure 11b compares the calculated values from the β equation with the Weibull fit values for a single mix; and Figure 11c illustrates the empirical trends of β as a function of T and P within the calibration range. Since the final equation does not include R, Figure 11c is used solely to describe the smooth variations within the T–P plane and does not represent the effect of the ash content.

#### 4.4.3. Low-Dimensional Empirical Freeze–Thaw Damage Model

By substituting the common shape parameter and scale parameter equations into the Weibull damage expression, we obtain a mix-proportion-driven low-dimensional empirical freeze–thaw damage model:

(18) $$\begin{array}{c} D\left(n,T,P\right)=1-\exp\left[-{\left(\frac{n}{\exp(5.8306-0.03822x_{T}-0.08684x_{P}-0.09932x_{P^{2}})}\right)}^{2.1403}\right] \end{array}$$

In the equation, D is the freeze–thaw damage degree defined from the relative dynamic elastic modulus, n is the cycle count, and T and P are the total mineral admixture replacement rate and PVA fiber volume content, respectively. The low-dimensional equation reconstructs relative damage trends only within the material system and calibrated T–P domain used in this study. Because R is not present in the final equation, the model cannot replace the experimental interpretation of the fly ash to slag powder ratio in Section 3.4.

Reducing the model dimension improves parameter identifiability and preserves training degrees of freedom in each LOMO-CV fold, but it also limits the ability to reproduce local performance peaks. Consequently, model appraisal must consider both the overall cross-validation error and mixture-specific failures rather than relying on in-sample R^2^. In this study, the mapping is treated as a localized empirical screening tool; it is not extrapolated across water to binder ratios, raw material sources, production batches, pore structures, or freeze–thaw regimes that were not represented in calibration.

## 5. Validation and Applicability Analysis of the Low-Dimensional Model

### 5.1. Leave-One-Mix Cross-Validation of Freeze–Thaw Nodes

**Figure 12:**
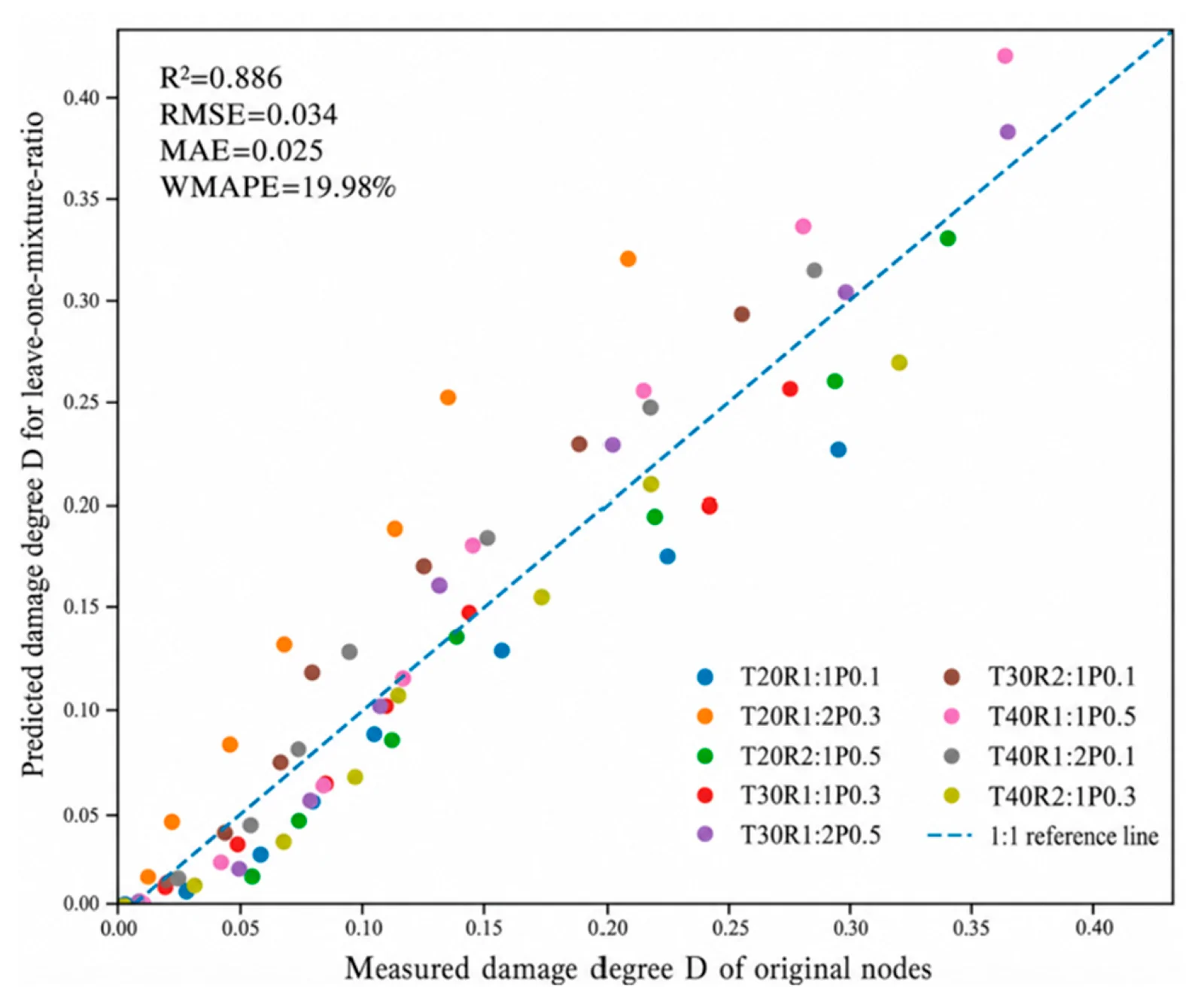
LOMO-CV predictions versus measured freeze–thaw damage nodes.

LOMO-CV was used to examine local interpolation to mixtures omitted from parameter estimation. In each fold, one complete mixture (all eight measured nodes from 25 to 200 cycles) was removed, the scale parameter equation was re-estimated using the remaining eight mixtures, and the omitted mixture was then reconstructed. Treating the mixture as the resampling unit prevents different freeze–thaw nodes from the same mixture from appearing in both training and validation sets and avoids pseudoreplication of the cycle nodes. The LOMO-CV predictions for the omitted-mixture damage nodes are shown in Figure 12.

**Figure 13:**
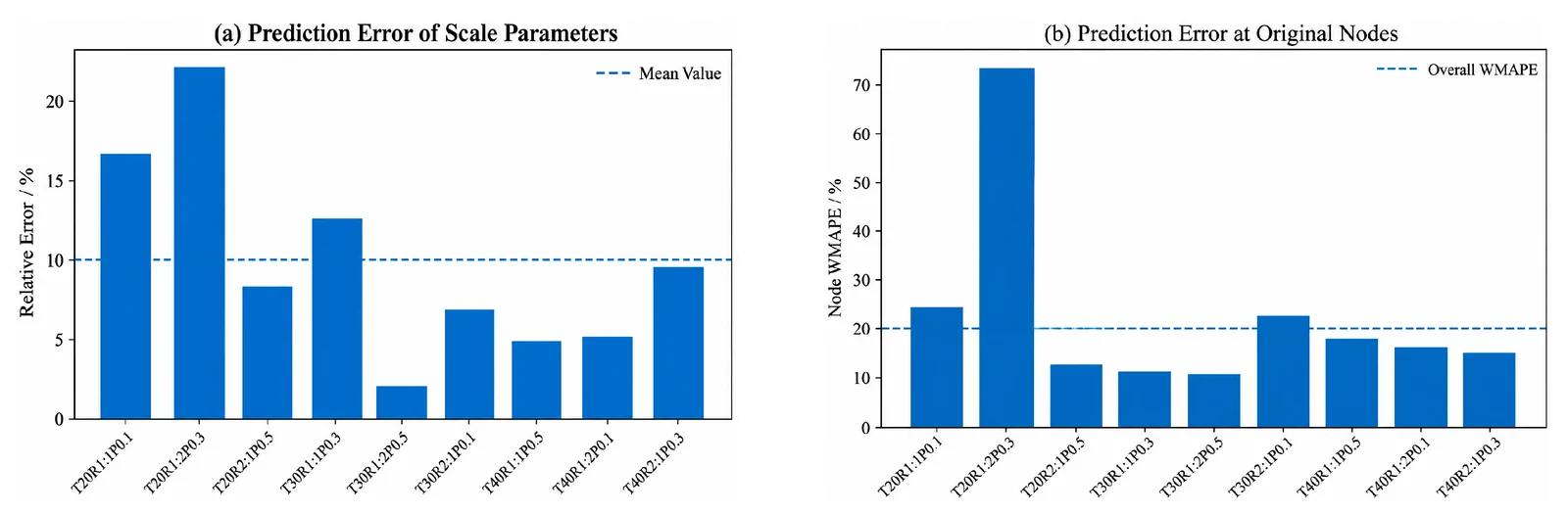
Mixture-by-mixture scale parameter and node errors in leave-one-mixture-out cross-validation.

Across the 72 omitted mixture nodes, the LOMO-CV predictions are distributed around the 1:1 line, with R^2^ = 0.886, RMSE = 0.034, MAE = 0.025, a maximum absolute damage difference of 0.117, and WMAPE = 19.98%. Conventional MAPE is 30.81% because early damage values close to zero amplify percentage errors; therefore, RMSE, MAE, and WMAPE are used as the primary cross-validation metrics. Mixture-specific errors are reported in Table 10 and Figure 13 so that local failures are not hidden by the overall statistics.

The mixture-specific results show a clear limitation: although most omitted mixtures have comparable errors, the low-dimensional model substantially underestimates the high-performing T20R1:2P0.3 mixture, for which the relative β error is 22.04%, the curve RMSE is 0.069, and the node WMAPE reaches 72.75%. Thus, the reduced order mapping preserves the overall response surface but does not reliably reproduce the local performance peak represented by the experimentally best mixture. It is suitable for trend comparison and preliminary screening within the calibrated range, but it should not be used to determine a precise critical freeze–thaw cycle count for a specific high-performance composition.

### 5.2. Local Validation Using Non-Orthogonal Mixes Within the Same Material System

**Figure 14:**
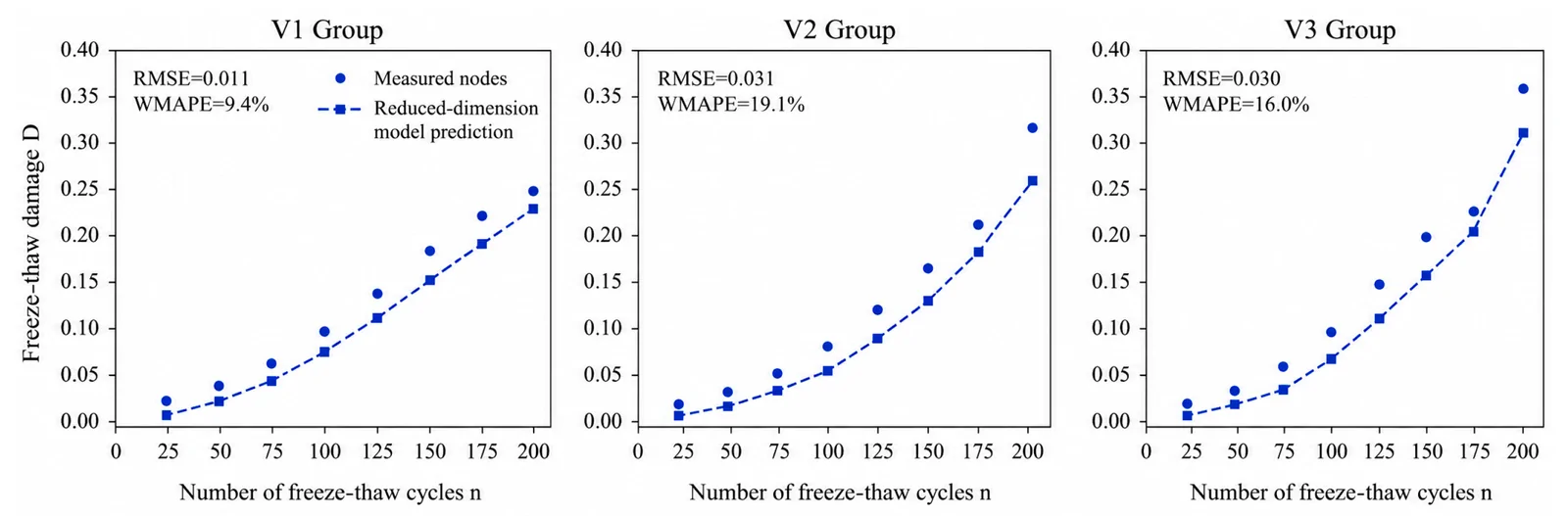
Local validation of non-orthogonal freeze–thaw damage nodes within the same material system.

Three non-orthogonal mixtures (V1–V3), not used in fitting the low-dimensional equation, were used for an additional local validation within the calibrated factor range. These mixtures used the same raw material sources, total binder content, water to binder ratio, preparation procedure, curing regimen, and freeze–thaw method as the modeling mixtures, while their factor levels avoided the L_9_ design points. The measured damage nodes were compared directly with the model-calculated curves. This design tests interpolation within the same material system; it is not an external validation across independent material sources or production batches. The mixture-specific validation results are summarized in Table 11. The measured and model-calculated damage curves for the three non-orthogonal validation mixtures are shown in Figure 14.

**Table 11:** Freeze–thaw node errors for the non-orthogonal local validation set.

Validation Mixture	β Predicted (Cycles)	β Fitted from Measured Nodes (Cycles)	Node R^2^	Node RMSE	Node MAE	Node WMAPE (%)
V1	347.1	354.7	0.984	0.011	0.009	9.36
V2	347.0	313.9	0.914	0.031	0.025	19.06
V3	312.1	288.6	0.940	0.030	0.025	15.99

For the 24 measured nodes from the three non-orthogonal validation mixtures, the overall R^2^ is 0.943, RMSE = 0.026, MAE = 0.020, WMAPE = 15.36%, and the maximum absolute damage difference is 0.056. V1 agrees closely with the measured nodes, whereas V2 and V3 show some underestimation in the middle to late stages. These results support a degree of local interpolation stability within the same material system and calibrated factor range. However, no independent raw material source or repeated production batch validation was available; therefore, these results cannot be used as evidence of external generalizability.

### 5.3. Model-Based Critical Cycle Estimate and Its Interpretation

Using a relative reduction in dynamic elastic modulus to 60% (i.e., D = 0.4) as the calculation threshold, solving Equation (18) in reverse yields the critical freeze–thaw cycle count:

(19) $$\begin{array}{c} N_{f}=\beta (T,P){\left[-\ln(0.6)\right]}^{\left(\frac{1}{\alpha 0}\right)} \end{array}$$

**Figure 15:**
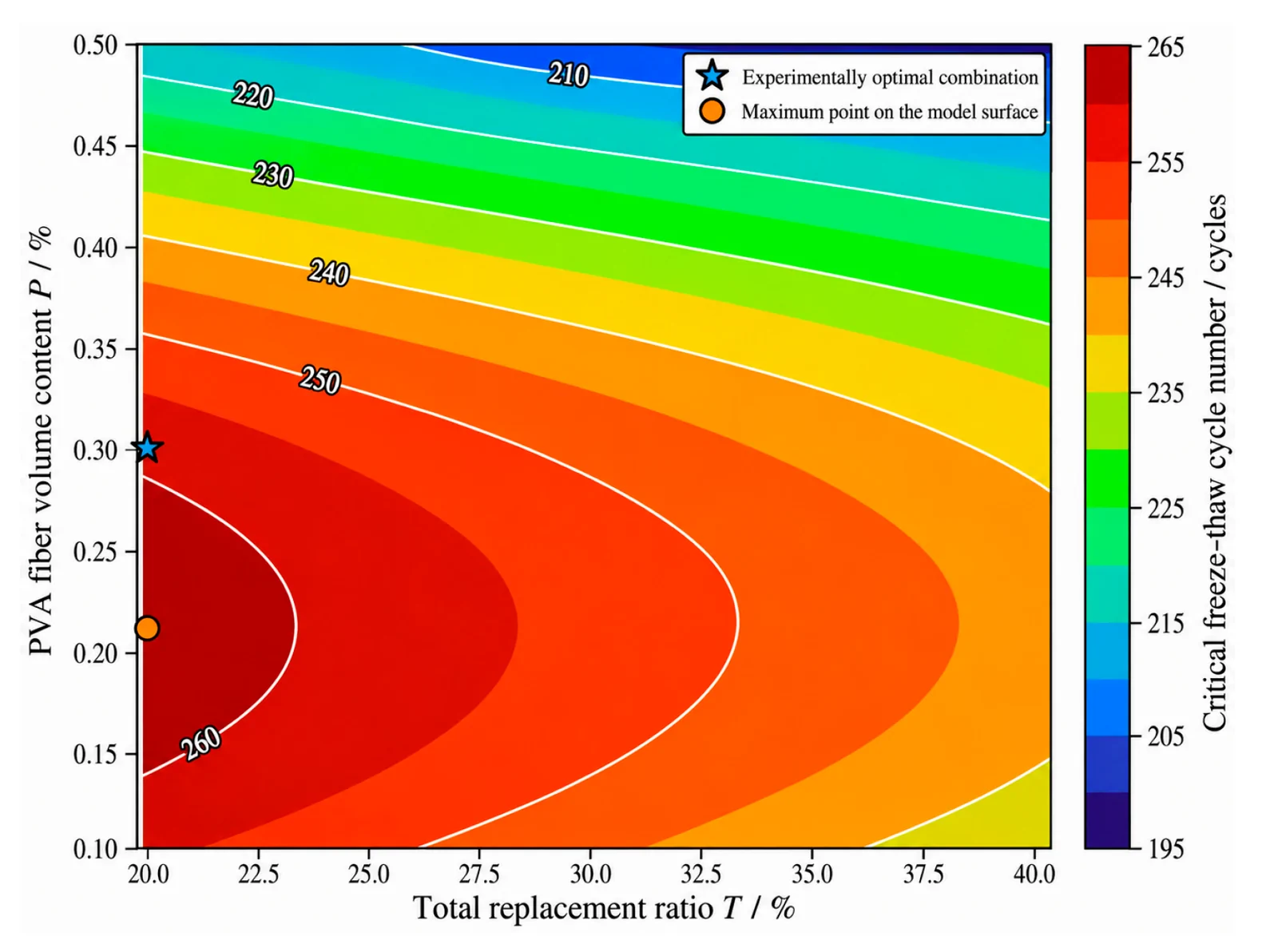
Model-based critical cycle indicator within the calibrated T–P domain.

Using Equation (19), the D = 0.4 threshold was evaluated only as a model-based critical cycle indicator over T = 20–40% and P = 0.1–0.5%. Figure 15 therefore represents the smoothed trend of the reduced order T–P mapping. Because R is absent from the final equation, the fly ash to slag powder ratio must still be selected from the measured orthogonal results rather than from this surface.

At the experimentally best factor combination T = 20% and P = 0.3%, the model-based D = 0.4 threshold occurs at approximately 259 cycles. The mathematical surface reaches a slightly higher maximum of approximately 263 cycles near T = 20% and P ≈ 0.21%. These two statements have different meanings: P = 0.3% is the best level supported directly by the orthogonal experiment, whereas P ≈ 0.21% is only the maximum of a smoothed reduced order surface. Because the experimental observations ended at 200 cycles, both 259 and 263 cycles are extrapolated mathematical estimates rather than measured frost resistance grades or validated service lives. The model surface maximum must therefore not be used to redefine the experimental optimum.

### 5.4. Applicability Domain and Limitations

The low-dimensional model is calibrated under a single water to binder ratio, consistent raw material sources, one preparation/curing protocol, and one rapid freeze–thaw regime. Material source and batch variability, air content characteristics, pore structure, saturation, and fiber dispersion state were not treated as independent variables. The final equation retains only T and P as stable predictors; this reflects the limited identifiability of continuous multi-factor effects with nine independent orthogonal mixtures and does not imply that R is physically unimportant. LOMO-CV and the three non-orthogonal mixtures remain within the same material system and therefore provide local interpolation evidence only. No repeated batch or cross-material external validation is claimed. The applicability domain and extrapolation status of the proposed low-dimensional empirical model are summarized in Table 12.

**Table 12:** Applicability domain and extrapolation status of the low-dimensional empirical model.

Interpretation Outside Domain	Calibrated/Validated Domain	Condition
Not validated outside this interval	20–40%	Total replacement rate T
Not validated outside this interval	0.1–0.5%	PVA fiber volume content P
Use Section 3.4. experimental trends; the low-dimensional model cannot optimize R	1:1, 1:2, and 2:1 experimentally investigated; R not retained in final mapping	Fly ash to slag ratio
Recalibration required for other ratios	0.45	Water to binder ratio
No claim of cross-source or batch-to-batch generalization	Same material sources as the modeling and local validation mixtures; batch variation not independently assessed	Raw material sources/batches
Values beyond 200 cycles are mathematical extrapolation	0–200 cycles	Freeze–thaw observations
Recalibration/validation required if conditions change	Procedures used in this study; rapid freeze–thaw testing according to GB/T 50082-2009	Preparation, curing, and test regime

Future work should add independent production batches and material sources, extend the factor combinations around the local high-performance region, and incorporate state variables such as air content characteristics, pore structure, saturation, fiber dispersion, and material reactivity before broader generalization is attempted.

## 6. Conclusions

(1) Under the tested material system and experimental conditions, the relative influence of the three factors on the relative dynamic elastic modulus after 200 freeze–thaw cycles followed the order PVA fiber volume content > total mineral admixture replacement rate > fly ash to slag powder mass ratio. Among the tested mixtures, T20R1:2P0.3 (20% total replacement, fly ash to slag powder ratio of 1:2, and 0.3% PVA fiber) exhibited the best freeze–thaw performance, retaining a relative dynamic elastic modulus of 79.18% after 200 cycles together with relatively limited surface deterioration and mass loss.
(2) The two-parameter Weibull function effectively describes the transition from slow early damage accumulation to accelerated later-stage degradation. In the original damage space, the average RMSE decreased from 0.032 for the classical exponential model to 0.019 for the Weibull model, while the average MAPE decreased from 5.07% to 2.98%. The R^2^ values from Weibull linearization are reported separately as transformed space regression statistics and are not treated as original space predictive metrics.
(3) With only nine independent mixtures available for parameter mapping, a common shape parameter α_0_ = 2.1403 and a low-dimensional lnβ equation were selected using AICc and LOMO-CV. The 72 omitted mixture nodes yielded R^2^ = 0.886, RMSE = 0.034, MAE = 0.025, and WMAPE = 19.98%; however, T20R1:2P0.3 exhibited a node WMAPE of 72.75%. The mapping therefore captures the overall response trend but does not reliably reproduce local high-performance peaks.
(4) The three non-orthogonal mixtures within the same material system yielded R^2^ = 0.943, RMSE = 0.026, and WMAPE = 15.36%, supporting local interpolation rather than external generalization across different material sources, water to binder ratios, or production batches. At T = 20% and P = 0.3%, the model-based D = 0.4 threshold was approximately 259 cycles; because the measured range ended at 200 cycles, this value represents mathematical extrapolation and should not be interpreted as a measured frost resistance grade or validated service life.

## Figures and Tables

**Table 1:** Chemical composition of cementitious materials (ω/100%).

Composition	SiO_2_	Al_2_O_3_	Fe_2_O_3_	CaO	MgO	K_2_O	SO_3_	Na_2_O
Cement	19.6	6.5	3.5	66.3	0.7	0.3	2.5	0.6
Fly ash	55.05	26.41	6.39	2.01	2.40	2.28	0.83	0.69
Slag powder	35.50	13.36	1.25	44.15	0.84	-	1.32	-

**Table 2:** Key technical specifications of polyvinyl alcohol fiber.

Tensile Strength (MPa)	Elongation at Break (%)	Specific Modulus (cN/dtex)	Equivalent Elastic Modulus (GPa)	Softening Point in Water (°C)	Density (g/cm^3^)	Alkali Resistance (%)
1400–1600	17 ± 3.0	380	35–39	114	0.91	98–100

Note: The two modulus entries describe the same fiber stiffness in supplier-specific and conventional engineering unit systems; “elongation at break” and “density” are used to avoid the previous ambiguous translations “dry fracture depth” and “linear density”.

**Table 3:** Physical properties of aggregates.

Aggregate Type	Particle Size Range /mm	Bulk Density /(kg/m^3^)	Apparent Density /(kg/m^3^)	Silt Content /%	Moisture Content /%
Coarse aggregate	5–20	1530	2690	1.5	0.01
Fine aggregate	0.15–5	1438	2565	1.3	3.5

**Table 4:** PVA-FA-SPC mix proportions.

No.	Total Replacement Rate (%)	Compound Proportion	PVA Dosage (%)	Cement (kg/m^3^)	Fly Ash (kg/m^3^)	Granulated Blast Furnace Slag (kg/m^3^)	Polyvinyl Alcohol (kg/m^3^)	Sand (kg/m^3^)	Stone (kg/m^3^)	Water (kg/m^3^)	Water-Reducing Agent (%)
T0R0P0	0	0:0	0.0	411	0	0	0	848	956	185	3.8
T20R1:1P0.1	20	1:1	0.1	328.8	41.1	41.1	1.26	848	956	185	3.8
T20R1:2P0.3	20	1:2	0.3	328.8	27.4	54.8	3.78	848	956	185	3.8
T20R2:1P0.5	20	2:1	0.5	328.8	54.8	27.4	6.3	848	956	185	3.8
T30R1:1P0.3	30	1:1	0.3	287.7	61.65	61.65	3.78	848	956	185	3.8
T30R1:2P0.5	30	1:2	0.5	287.7	41.1	82.2	6.3	848	956	185	3.8
T30R2:1P0.1	30	2:1	0.1	287.7	82.2	41.1	1.26	848	956	185	3.8
T40R1:1P0.5	40	1:1	0.5	246.6	82.2	82.2	6.3	848	956	185	3.8
T40R1:2P0.1	40	1:2	0.1	246.6	54.8	109.6	1.26	848	956	185	3.8
T40R2:1P0.3	40	2:1	0.3	246.6	109.6	54.8	3.78	848	956	185	3.8

**Table 5:** Design of orthogonal experiments with factors and levels.

Factor Number	Factor Name	Level 1	Level 2	Level 3
A	Total replacement rate for blended cement (by cement mass)	20%	30%	40%
B	Blending ratios for fly ash and slag powder	1:1	1:2	2:1
C	Volume content of PVA fibers	0.1%	0.3%	0.5%

**Table 6:** Orthogonal experiment design and measured results of relative dynamic modulus of elasticity.

Specimen Number	Combination of Factor Levels	A Total Replacement Ratio	B Blending Ratio	C Fiber Content	Relative Dynamic Elastic Modulus After 200 Freeze–Thaw Cycles/%
T20R1:1P0.1	A1B1C1	20%	1:1	0.1%	70.50
T20R1:2P0.3	A1B2C2	20%	1:2	0.3%	79.18
T20R2:1P0.5	A1B3C3	20%	2:1	0.5%	66.00
T30R1:1P0.3	A2B1C2	30%	1:1	0.3%	72.50
T30R1:2P0.5	A2B2C3	30%	1:2	0.5%	63.50
T30R2:1P0.1	A2B3C1	30%	2:1	0.1%	74.50
T40R1:1P0.5	A3B1C3	40%	1:1	0.5%	63.60
T40R1:2P0.1	A3B2C1	40%	1:2	0.1%	71.50
T40R2:1P0.3	A3B3C2	40%	2:1	0.3%	68.00
T0R0P0	-	0	0:0	0	50.61

**Table 7:** Exploratory main effect sum-of-squares decomposition for the relative dynamic elastic modulus after 200 freeze–thaw cycles.

Source	SS	df	SS/Total SS (%)
A: Total mineral admixture replacement rate	26.65	2	12.31
B: Fly ash to slag powder mass ratio	10.37	2	4.79
C: PVA fiber volume content	140.46	2	64.90
Residual	38.94	2	17.99
Total	216.42	8	100.00

Note: The analysis was performed using one retained mean response for each orthogonal mixture. Because specimen-level replicate records are unavailable for estimating pure experimental error, the residual term should not be interpreted as an independent estimate of experimental variance. The sum-of-squares proportions are therefore used only as exploratory measures of relative effect magnitude.

**Table 8:** Weibull linearized regression parameters and original damage space fit metrics.

No.	a	b	R^2^_ln–ln	R^2^_D	RMSE_D
T0R0P0	2.1134	−11.5639	0.974	0.983	0.021
T20R1:1P0.1	2.1243	−12.2794	0.973	0.967	0.017
T20R1:2P0.3	2.2129	−13.2627	0.995	0.986	0.008
T20R2:1P0.5	2.1014	−11.8554	0.939	0.954	0.024
T30R1:1P0.3	2.2367	−12.8656	0.980	0.954	0.020
T30R1:2P0.5	2.0692	−11.7106	0.952	0.965	0.022
T30R2:1P0.1	2.1624	−12.6859	0.967	0.945	0.019
T40R1:1P0.5	2.1145	−11.9094	0.960	0.960	0.023
T40R1:2P0.1	2.1388	−12.4209	0.974	0.961	0.018
T40R2:1P0.3	2.1021	−12.0797	0.963	0.954	0.021

**Table 9:** Coefficients of the Weibull probability distribution damage model.

No.	α	β	Fitting Formula
T0R0P0	2.1134	237.865	$D(n)=1-exp\left[-{\left(\frac{n}{237.865}\right)}^{2.1134}\right]$
T20R1:1P0.1	2.1243	323.903	$D(n)=1-exp\left[-{\left(\frac{n}{323.903}\right)}^{2.1243}\right]$
T20R1:2P0.3	2.2129	400.758	$D(n)=1-exp\left[-{\left(\frac{n}{400.758}\right)}^{2.2129}\right]$
T20R2:1P0.5	2.1014	281.932	$D(n)=1-exp\left[-{\left(\frac{n}{281.932}\right)}^{2.1014}\right]$
T30R1:1P0.3	2.2367	314.834	$D(n)=1-exp\left[-{\left(\frac{n}{314.834}\right)}^{2.2367}\right]$
T30R1:2P0.5	2.0692	287.000	$D(n)=1-exp\left[-{\left(\frac{n}{287.000}\right)}^{2.0692}\right]$
T30R2:1P0.1	2.1624	353.041	$D(n)=1-exp\left[-{\left(\frac{n}{353.041}\right)}^{2.1624}\right]$
T40R1:1P0.5	2.1145	279.291	$D(n)=1-exp\left[-{\left(\frac{n}{279.291}\right)}^{2.1145}\right]$
T40R1:2P0.1	2.1388	332.758	$D(n)=1-exp\left[-{\left(\frac{n}{332.758}\right)}^{2.1388}\right]$
T40R2:1P0.3	2.1021	313.090	$D(n)=1-exp\left[-{\left(\frac{n}{313.090}\right)}^{2.1021}\right]$

**Table 10:** Mixture-specific errors from leave-one-mixture-out cross-validation.

Mix Proportions	β Prediction Error (%)	Curve RMSE	Curve MAE	Curve WMAPE/%	Maximum Absolute Difference
T20R1:1P0.1	16.40	0.035	0.028	23.77	0.068
T20R1:2P0.3	22.04	0.069	0.055	72.75	0.117
T20R2:1P0.5	8.55	0.023	0.020	12.72	0.034
T30R1:1P0.3	12.51	0.018	0.014	11.77	0.042
T30R1:2P0.5	2.23	0.020	0.017	10.97	0.029
T30R2:1P0.1	7.01	0.029	0.022	22.71	0.046
T40R1:1P0.5	5.07	0.035	0.028	18.04	0.057
T40R1:2P0.1	5.35	0.023	0.019	16.75	0.034
T40R2:1P0.3	9.62	0.025	0.020	15.40	0.051

## Data Availability

The original group-level freeze–thaw cycle node data and the minimum dataset supporting the principal experimental and model analysis results are provided in Supplementary File S1. Further inquiries can be directed to the corresponding author.

## Supplementary Materials

The following supporting information can be downloaded at: https://www.mdpi.com/article/10.3390/ma19173767/s1.
